# TFIIB-Related Protein BRP5/PTF2 Is Required for Both Male and Female Gametogenesis and for Grain Formation in Rice

**DOI:** 10.3390/ijms242216473

**Published:** 2023-11-18

**Authors:** Guangna Chen, Hongliang Hu, Xinhui Chen, Jialuo Chen, Siyi Wang, He Ning, Cheng Zhu, Su Yang

**Affiliations:** 1 College of Life Sciences, China Jiliang University, 258 Xueyuan Street, Hangzhou 310018, China; s21090710003@cjlu.edu.cn (G.C.); hlhu2020@163.com (H.H.); s21090710005@cjlu.edu.cn (X.C.); s20090710003@cjlu.edu.cn (J.C.); S23090710053@cjlu.edu.cn (S.W.); pzhch@cjlu.edu.cn (C.Z.); 2 Institute of Crop Science and Zhejiang Key Laboratory of Crop Germplasm, Zhejiang University, Hangzhou 310058, China

**Keywords:** rice, transcription factor, TFIIB, gametogenesis, pollen development

## Abstract

Transcription factor IIB (TFIIB) is a general transcription factor for RNA polymerase II, exerting its influence across various biological contexts. In the majority of eukaryotes, TFIIB typically has two homologs, serving as general transcription factors for RNA polymerase I and III. In plants, however, the TFIIB-related protein family has expanded greatly, with 14 and 9 members in Arabidopsis and rice, respectively. BRP5/pollen-expressed transcription factor 2 (PTF2) proteins belong to a subfamily of TFIIB-related proteins found only in plants and algae. The prior analysis of an *Arabidopsis atbrp5* mutant, characterized by a T-DNA insertion at the 5′ untranslated region, demonstrated the essential role of BRP5/PTF2 during the process of pollen germination and embryogenesis in *Arabidopsis*. Using a rice transformation system based on CRISPR/Cas9 technology, we have generated transgenic rice plants containing loss-of-function frameshift mutations in the *BRP5/PTF2* gene. Unlike in the *Arabidopsis atbrp5* mutant, the *brp5/ptf2* frameshift mutations were not transmitted to progeny in rice, indicating an essential role of BRP5/PTF2 in both male and female gamete development or viability. The silencing of rice *BRP5/PTF2* expression through RNA interference (RNAi) had little effect on vegetative growth and panicle formation but strongly affected pollen development and grain formation. Genetic analysis revealed that strong RNAi silencing of rice BRP5/PTF2 was still transmissible to progeny almost exclusively through female gametes, as found in the *Arabidopsis atbrp5* knockdown mutant. Thus, reduced rice *BRP5/PTF2* expression impacted pollen preferentially by interfering with male gamete development or viability. Drawing upon these findings, we posit that *BRP5/PTF2* assumes a distinct and imperative function in the realm of plant sexual reproduction.

## 1. Introduction

In eukaryotic organisms, the transcription of the nuclear genome is accomplished through the utilization of a trinity of multi-subunit RNA polymerase enzymes, namely Pol I, II, and III [1]. Of the three RNA polymerases, Pol II synthesizes messenger RNA (mRNA), microRNA (miRNA), and small nuclear RNA (snRNA), which have been extensively investigated in scientific research. The initiation of Pol II transcription involves a multitude of seven distinct general transcription factors (TATA box-binding protein or TBP, transcription factor IIA or TFIIA, TFIIB, TFIID, TFIIE, TFIIF, and TFIIH) [1,2]. During the initiation process, these general transcription factors discern promoter elements that assist and recruit Pol II in the DNA double-strand opening and RNA synthesis at the initial stage. The spatial and temporal regulation of Pol II-mediated gene-specific transcription of genes usually requires specific transcription factors that govern the process of gene transcription by identifying particular cis-acting DNA elements within target genes and recruit co-activators or co-repressors, such as the mediator complex, P3000, and general transcription factors, to facilitate or restrain transcription [3]. Gene-specific transcription factors can recruit proteins that either mobilize or chemically modify nucleosomes at target genes, thereby facilitating or inhibiting the accessing of the transcription apparatus and its regulators, thus contributing to transcriptional control.

In eukaryotic organisms, numerous gene-specific transcription factors regulate intricate tissue- and cell-specific gene expression [1]. Some gene-specific transcription factors belong to large families containing many members with both shared and distinct functions. Conversely, general transcription factors, essential for the transcription of all active genes, typically have small families with only 2–4 members. For instance, Pol I and III, responsible for synthesizing 25S ribosomal RNA (rRNA) and small untranslated RNAs (tRNA and 5S rRNA), also rely on these same or similar general transcription factors for transcription initiation [1]. For example, non-plant eukaryotes typically possess 3 or 4 TFIIB-related proteins that function as general transcription factors. In yeast (*Saccharomyces cerevisiae*), TFIIB acts as a general transcription factor for Pol II, while two counterparts of TFIIB, Rrn7 and Brf-1, serve as general transcription factors for Pol I and Pol III, respectively [4]. Intriguingly, within the realm of plants, there are a large number of TFIIB-like proteins, comprising a total of 14 and 9 members in Arabidopsis and rice, respectively [4]. In contrast, there are only 2–4 members in other general transcription factor families in *Arabidopsis* [4]. Henceforth, the TFIIB-like protein family has undergone expansion within the plant kingdom, resulting in potentially redundant, specialized, and diverse functions within this particular set of general transcription factors, particularly in the context of plant growth, development, and stress responses.

During transcription, TFIIB interacts with both Pol II and TBP, playing a pivotal role in selecting transcription start sites, initiating promoter opening, and ensuring subsequent clearance. Eukaryotic TFIIB is structurally, functionally, and evolutionarily related to bacterial sigma (s) factor and archaeal transcription factor B (TFB); both have an expanded family for the coordinated transcription of discrete sets of genes [5]. While there are many studies into TFIIB in non-plant eukaryotes, the research in plants has been relatively limited, with the majority of studies focused on the model plant *Arabidopsis* [6]. *Arabidopsis AtTFIIB1* and *AtTFIIB2* encoded a pair of structurally analogous TFIIB-like proteins, each exhibiting unique expression patterns in the endosperm and mature pollens [7]. Mutations in *AtTFIIB1* could influence the growth and guidance of pollen tubes, as well as the reception of fertilization, and the development of the endosperm [7]. The employment of the *AtTFIIB1* promoter to propel the expression of AtTFIIB2 demonstrates its capacity to completely alleviate the deficiencies in pollen growth, guidance, and reception while offering partial alleviation of the defect in endosperm development in the *attfiib1* mutants [7]. This observation suggests that these two TFIIB-like proteins have undergone functional divergence in part through distinct expression patterns. The defects of the *attfiib1* mutants in endosperm development could also be partially rescued by *AtBRP2* when driven by the *AtTFIIB1* promoter [7]. *AtBRP2* encodes a TFIIB-related protein in *Arabidopsis* with close homologs found only in the Brassicaceae family [7]. AtBRP4, a protein exhibiting high similarity to TFIIB assumes an essential role in the progression of the cell cycle within the male gametophytes [8]. AtMEE12 bears a structural resemblance to the TFIIB-like Rrn7 (yeast) and TAF1B (human), both of which function as general transcription factors for Pol I [9]. *AtMEE12* exhibits a robust expression in all *Arabidopsis* tissues, with the exception of leaves and stems. A transposon-tagged mutant for *AtMEE12* was zygotically lethal and also defective in the guidance of pollen tubes [9]. Interestingly, subsequent analysis revealed that *AtMEE12* regulates pollen tube attraction by acting as a TFIIB-esque general transcription factor for Pol II and interacting with other transcription regulators to direct the expression of a subset of central cell-specific genes [10]. There are three *Arabidopsis* proteins (AtBRF1, 2, and 3) that have shown significant homology with yeast and human BRF and act as Pol III general transcription factors [11]. *Arabidopsis* T-DNA insertion single mutants for three BRF genes as well as *atbrf1/3* and *atbrf2/3* double mutants have exhibited normalcy in both the growth and developmental processes. However, the *atbrf1/2* double mutant has proven lethal [11]. Further analysis showed that mutations in both *AtBRF1* and *AtBRF2* cause the abortion of large numbers of macrogametes and microgametes, ultimately leading to a complete failure in zygote generation. Thus, a majority of the analyzed *Arabidopsis* TFIIB-like proteins assume pivotal functions in specific reproductive processes [11].

Phylogenetic analysis has revealed that the expanded TFIIB-like protein family in plants can be categorized into five distinct subfamilies, with the majority of plant TFIIB-like proteins falling into three major subfamilies corresponding with the TFIIB, Rrn7/TAF1B/MEE12, and Brf clades found in all eukaryotes [4]. Plants also possess two additional subfamilies of TFIIB-associated proteins, denoted as BRP1 and BRP5, which are absent from other eukaryotic organisms. In *Arabidopsis*, there is a single member of the BRP5 subfamily (AtBRP5), which was also designated as pollen-expressing transcription factor 2 (PTF2) owing to its manifestation in the maturation process of pollen grains [12]. RT-qPCR analysis, along with β-glucuronidase (GUS) or green fluorescent protein (GFP) fusion constructs, further confirms the expression of AtBRP5 in various tissues, including inflorescence, developing pollen grains, and embryos [12]. Moreover, it was also expressed in shoot apical meristems, root tips, and lateral root primordia, while being absent from leaves [12]. In developing pollen grains, it was first expressed in the vegetative nuclei of the pollen grains in the early binucleate stage. This expression persisted throughout the second pollen mitosis but experienced a notable decline during the late trinucleate developmental stage [12]. No discernible expression was found in the germinating pollen grains or the pollen tubes, nor in mature pollen grains released from anthers [12]. Consequently, *AtBRP5* was mainly expressed in developing pollen grains and tissues characterized by vigorous cellular proliferation and differentiation.

A mutant of *AtBRP5* characterized by a T-DNA insertion in the 5’ untranslated region (5’-UTR) had been identified; however, homozygous mutant plants could not be obtained [12]. Genetic hybridization with wild-type plants revealed that transmission of the *atbrp5* mutation through the male gametophyte was significantly reduced, while the impact on female gametophytic function was relatively minor [12]. Further analysis unveiled that the mutation engenders a failure in the germination of pollen. Pollen-rescue experiments have elucidated that the mutation additionally disrupts embryogenesis, leading to seed abortion [12]. AtBRP5 protein engaged in interaction with TBP2 and exhibited binding affinity toward the double-stranded DNA (dsDNA) [12]. In addition, AtBRP5 had the capability to form a homodimeric structure, enabling it to engage in intricate interactions with the subunits of RNA polymerases. These findings strongly confirm that AtBRP5 has a pivotal role in the processes of *Arabidopsis* pollen germination and embryogenesis [12]. However, these reported roles of *Arabidopsis* AtBRP5/PTF2 were based on an *Arabidopsis* mutant with a T-DNA inserted in the 5′-UTR of the AtBRP5/PTF2 gene, which is likely to be a knockdown mutant and, thus, may not fully reveal its biological functions.

Despite the widespread expansion of the TFIIB-like protein family across various plant species, the functional investigation of this protein family has predominantly focused on *Arabidopsis*, with limited exploration of other plant species [6]. Given the extensive diversity of plants that have a wide range of traits during growth and development, it will be of profound interest to ascertain the structural and functional preservation and divergence of the expanded TFIIB-like factors among diverse botanical species. Here, we report on the functional analysis of the rice homolog of *Arabidopsis* AtBRP5/PTF2. Using CRISPR/Cas9, we have generated transgenic rice plants containing loss-of-function frameshift mutations in the *OsBRP5/PTF2* gene and discovered that the mutations were not transmitted to progeny in rice. Therefore, OsBRP5/PTF2 is required for the development or survival of male and female gametes. The silencing of rice *BRP5/PTF2* expression had little effect on vegetative growth and panicle formation but strongly affected pollen development and grain formation, as found in the Arabidopsis *atbrp5* knockdown mutant. These results substantiate the pivotal and distinct function of plant-specific BRP5/PTF2 in the realm of botanical reproductive processes.

## 2. Results

### 2.1. OsBRP5 Protein Sequence

**Figure 1:**
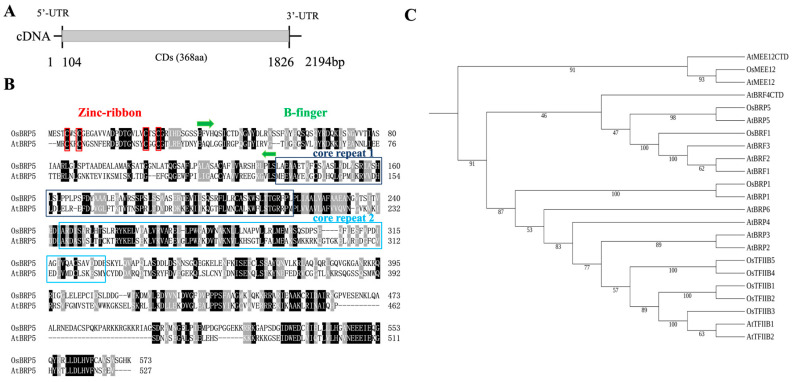
Rice BRP5 gene structure and protein sequence. (**A**) The OsBRP5 gene (LOC_Os08g42020) full-length cDNA structure. (**B**) Amino acid sequence alignment of Arabidopsis and rice BRP5 proteins. Identical and similar amino acids are highlighted in black and gray, respectively. The red box indicates the positions of the four conserved cysteine bases in the zinc ribbon domain. The green arrows indicate the conserved B-finger domain. The dark blue and light blue boxes indicate the two repeat core domains. (**C**) A phylogenetic tree of the TFIIB-related proteins from Arabidopsis and rice. A phylogenetic tree was constructed using each gene’s protein sequence with MEGA 6.0 software.

In the present investigation, we retrieved the OsBRP5 gene from the rice genome database, employing in silico analysis. Similar to Arabidopsis, the rice genome harbors a solitary OsBRP5 gene (LOC_Os08g42020). Both Arabidopsis and rice BRP5 genes are single-exon genes with no intron. The OsBRP5 gene was cloned from the japonica rice (*Oryza sativa* Nipponbare var.) via PCR. The OsBRP5 gene full-length cDNA contains a 5′-UTR of 104 bp, a 3′-UTR of 368 bp with a poly (A) tail, and a CDS of 1722 bp that encodes a 573 amino acid polypeptide (Figure 1A). The protein’s MW is estimated to be 61.82 kDa, while its theoretical pI is 6.09.

Sequence analysis revealed that the OsBRP5 protein exhibits a zinc ribbon domain at its N-terminal, as well as two cyclin fold core domains (Figure 1B). Based on its characteristics, the protein can be classified as being within the TFIIB superfamily (Figure 1B). A BLASTP search unveiled a strong structural homology between the rice OsBRP5 protein and the Arabidopsis AtBRP5 protein (Figure 1B). Furthermore, it was found that AtBRP5 and OsBRP5 had a high sequence similarity in the C-terminal region. Upon prediction of conserved domains within the sequences, it was observed that the C-terminal regions exhibited an absence of conserved domains. Meanwhile, Niu et al. also found that there was no conserved domain in the C-terminal of Arabidopsis BRP5 [12]. Phylogenetic analysis showed that the TFIIB gene family in rice and Arabidopsis could be divided into three subfamilies (Figure 1C). OsBRP5 and AtBRP5 could be clustered into the same branch at the end of the phylogenetic tree, indicating that they had a high homology. This suggests that BRP5 is relatively conserved during evolutionary development across plant species. Furthermore, it is worth noting that the OsBRP5 protein also exhibits a marked divergence in sequence similarity from other general transcription factors such as TFIIB-related proteins in the rice genome (Figure 1C).

### 2.2. Expression and Subcellular Localization of OsBRP5

**Figure 2:**
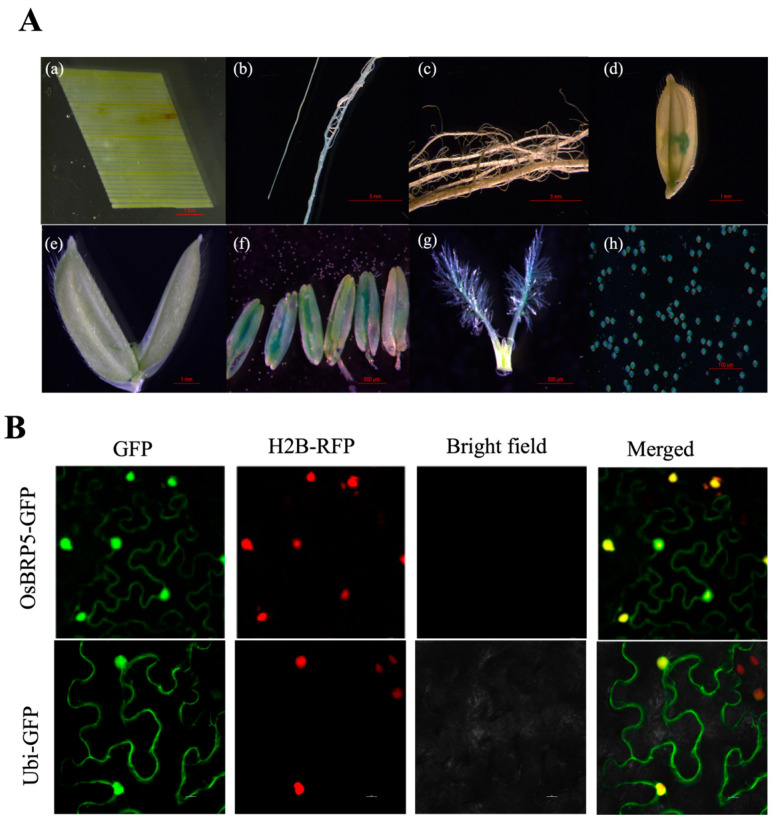
Analysis and subcellular localization of BRP5 expression in rice. (**A**) Expression activities of transgenic rice OsBRP5-GUS in different tissues and organs. Leaf blade (**a**); radicle (**b**); matured root (**c**); flower (**d**), glume (**e**); stamen (**f**); and pollen grains (**g**). (**B**) OsBRP5 subcells were localized in the epidermal cells of *N. benthamiana*, OsBRP5-GFP fusion, or GFP co-expressed with the nuclear marker H2B-RFP alone, and the signal was observed using a fluorescence microscope. Green fluorescence shows GFP, red fluorescence shows nucleus fluorescence, and yellow fluorescence shows the merged fluorescence. Bars = 20 µm.

To elucidate the expression profile of OsBRP5, we initiated our investigation by scrutinizing the expression data of OsBRP5 in the comprehensive rice expression database (http://bar.utoronto.ca/ (accessed on 21 September 2022)). As shown in Figure S1, OsBRP5 is mainly expressed in reproductive organs. To verify this result, the OsBRP5 promoter sequence (1600 bp) was isolated from rice genomic DNA, and a promoter-GUS fusion was constructed. The vector was introduced into the rice calli via Agrobacterium-mediated transformation. GUS staining in different tissues of OsBRP5-GUS transgenic plants was cultivated under optimal growth conditions and subsequently observed. The results showed that OsBRP5 was strongly expressed in reproductive organs such as flowers, pistils, stamens, and pollen grains (Figure 2A(d–h)). Among them, the expression of OsBRP5 in pistils and pollen grains was higher than in immature spikelets and stamens. We also noticed that OsBRP5 was seldom expressed in other parts, such as leaves, roots, stems, or seeds (Figure 2A(a–c)). This suggests that OsBRP5 may be involved in plant reproductive growth (Figure 2A), which is inconsistent with the predicted expression pattern from the rice expression database.

To determine the subcellular localization of the OsBRP5 protein in plant cells, an open reading frame of OsBRP5, lacking a stop codon, was fused with GFP controlled by the Maize Ubiquitin promoter. The construct was then used to transfuse *Nicotiana benthamiana* leaf. Transient expression analysis showed that OsBRP5-GFP fusion protein and RFP-H2B protein, a known nuclear marker, were co-located in the nucleus of *N. benthamiana* leaves 36 h after infection by Agrobacterium tumefaciens, while the *N. benthamiana* leaves transfected with GFP empty construct was localized in the cell nucleus and cell membrane (Figure 2B). This result suggested that OsBRP5 might still function as a nucleus-localized general transcription factor.

### 2.3. OsBRP5 Interacts with OsTBPs In Vitro and In Vivo

**Figure 3:**
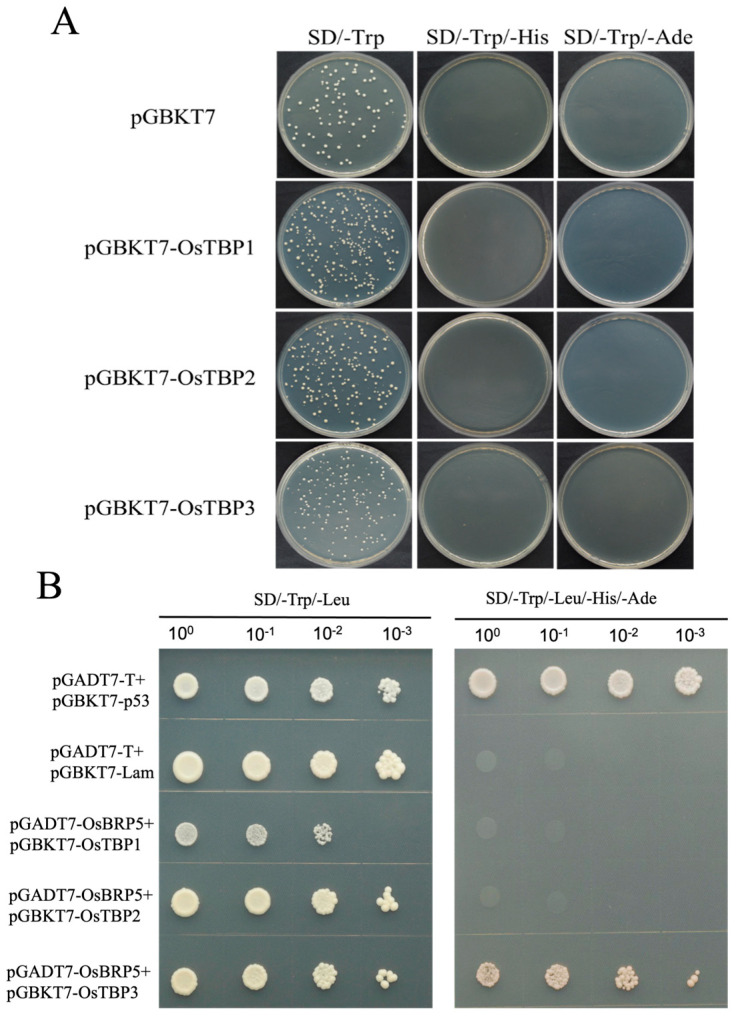
OsBRP5 interacted with OsTBP. (**A**) pGBKT7-OsTBP1/2/3 does not cause autoactivation or toxicity when introduced in yeast. (**B**) Yeast two-hybrid assays of OsBRP5 proteins with OsTBP3. Prey AD, the Gal4 activation domain fusion; bait BD, the Gal4 DNA-binding domain fusion. AD-T and BD-p53, the positive control; AD-T and BD-Lam, the negative control in the Y2H assay. The indicated fusion bait and prey constructs were co-transformed into yeast cells. The transformed yeast was cultured with SD/-Leu-Trp medium, and SD/-Leu-Trp-His-Ade was selected as the medium.

Gene regulation heavily relies on Pol II-mediated transcription, which is supported by seven general transcription factors including TFIIA, TFIIB, TFIID, TFIIE, TFIIF, TFIIH, and TBP. These general transcription factors require binding with Pol II and DNA to collectively form the preinitiation complex (PIC), enabling transcription [13]. To ascertain whether OsBRP5 functions as a general transcription factor or has evolved into a gene-specific transcription factor, we performed a yeast two-hybrid (Y2H) analysis to determine whether OsBRP5, as the bait, interacts with the prey, OsTBP, or not. The yeast two-hybrid system was employed to construct yeast vectors, namely, pGADT7-OsBRP5, pGBKT7-OsTBP1/2/3, positive control (pGADT7-T, and pGBKT7-p53), and negative control (pGADT7-T and pGBKT7-Lam). Our results showed that the introduction of pGBKT7-OsTBP1/2/3 into yeast does not give rise to autoactivation or toxicity (Figure 3A). Subsequently, OsBRP5 was co-transferred into yeast cells AH109 and coated on SD/-Trp-Leu and SD/-Ade/-His/-Leu/-Trp selective media, respectively. The results showed that the prey AD-OsBRP5 protein interacted with the bait BD-OsTBP3; however, there was no interaction between the prey AD-OsBRP5 and the bait BD-OsTBP1/2 (Figure 3B).

**Figure 4:**
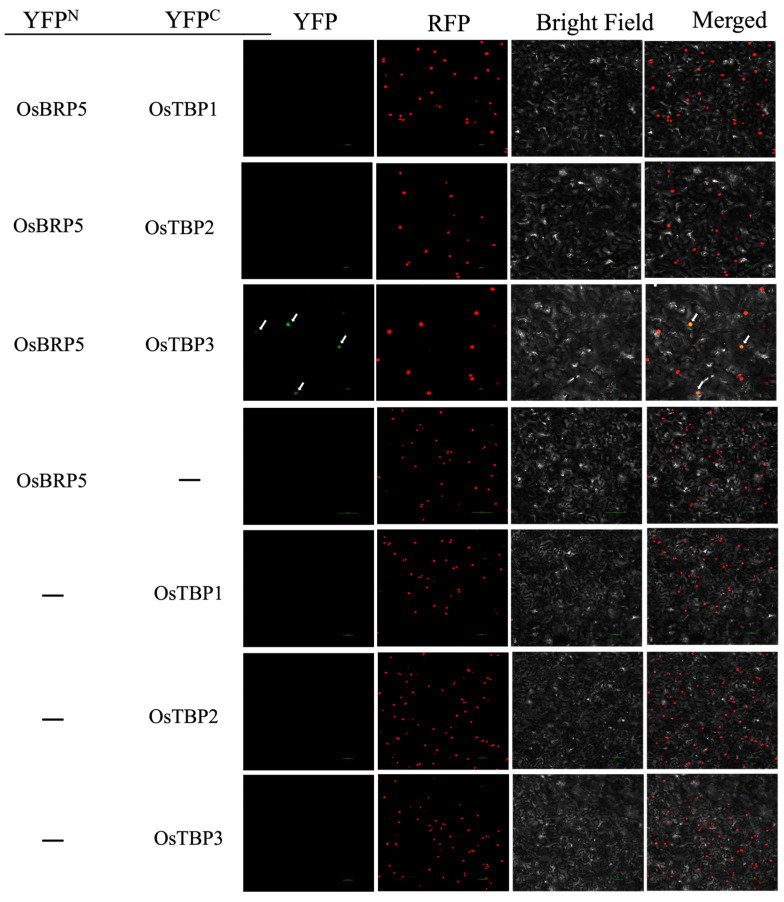
BiFC analysis of OsBRP5 and OsTBP interaction in *N. benthamiana* leaves. OsTBP 1/2/3 (OsTBP1/2/3-N-YFP) fused with the n terminal of YFP and OsBRP5 fused with the c terminal of YFP (OsBRP5-N-YFP) were infected and co-expressed in *N. benthamiana* for 48 h. YFP fluorescence (green), RFP autofluorescence (red), bright field, and combination images were taken using confocal microscopy. Bars = 50 μm.

To further confirm the potential interaction between OsBRP5 and OsTBP in plant cells, we conducted (BiFC) assays on Agrobacterium-infiltrated *N. benthamiana* plants. We fused the rice OsTBP to the N-terminal YFP (OsTBP1/2/3-N-YFP) and OsBRP5 to the C-terminal YFP fragment (OsBRP5-C-YFP) in the leaves of *N. benthamiana*. In control experiments where *N. benthamiana* was co-infected with OsBRP5-N-YFP and unfused C-YFP, no YFP fluorescence was produced (Figure 4). Likewise, the co-expression of OsTBP1-C-YFP, OsTBP2-C-YFP, and OsTBP3-C-YFP with unfused N-YFP also resulted in the absence of YFP fluorescence (Figure 4). When fused OsTBP3-N-YFP was co-expressed with OsBRP5-C-YFP in *N. benthamiana* leaves, YFP fluorescence was detected (Figure 3). When fused OsTBP1/2-N-YFP was co-expressed with OsBRP5-C-YFP in *N. benthamiana* leaves, YFP fluorescence was undetectable (Figure 4). These results showed that OsBRP5 does not interact with OsTBP1 or OsTBP2 anymore but still interacts with OsTBP3, which is in accordance with the results for Y2H. These findings further indicate that OsBRP5 may still function as a general transcription factor in the nucleus.

### 2.4. Generation and Characterization of Rice OsBRP5 Mutants

**Figure 5:**
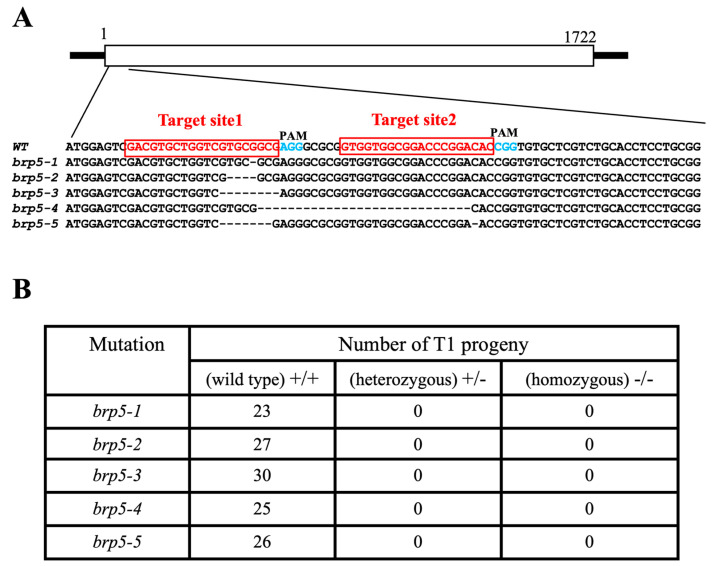
Targeted mutations of the rice OsBRP5 gene using CRISPR/Cas9 genome editing. (**A**) Comparison of OsBRP5 CDS in WT plants and five osbrp5 mutants. The red box represents the target site, and the blue color indicates PAM. (**B**) Identification results of five *osbrp5* mutants.

In order to elucidate the biological function of OsBRP5, we attempted to disrupt the OsBRP5 gene with the CRISPR/Cas9 genome-editing system. We designed two separate guide RNA sequences within the exon of OsBRP5 as the editing targets (Figure 5). After the successful construction of the CRISPR/Cas9 single-gene dual-target vector, Agrobacterium-mediated transformation was performed to obtain transgenic seedlings. Subsequently, mutation types were identified via PCR and Sanger sequencing. Among the 128 transgenic plants in the T_0_ generation, there are nine heterozygous frame-shift mutations (five different types) (Figure 5), six heterozygous non-frame-shift mutations (two had mutations in the zinc-ribbon domain, while the other four did not), and 113 WT plants (osbrp5^+/+^). No homozygous mutant (osbrp5^−/−^) was found in the T_0_ generation. The growth and developmental phenotypes, as well as the seed yield, of all CRISPR lines were observed, and no significant differences were found among brp5-1~5 lines, non-frame-shift lines, and WT plants.

Subsequently, we harvested the self-pollinated seeds from the T_0_ generation and observed the segregation pattern in the T1 generation. The results showed that all plants in the T_1_ generation were WT plants (osbrp5^+/+^). No homozygous plants (osbrp5^−/−^) or heterozygous plants (osbrp5^+/−^) were found. The sequencing results showed that the parental frameshift chain could not be inherited by the next generation. Together, these results show that OsBRP5 exhibits a homozygous lethal phenomenon (Figure 5), indicating that OsBRP5 might have very important biological functions. Hence, in contrast to its Arabidopsis counterpart AtBRP5, rice OsBRP5 is indispensable for optimal growth and viability, even under favorable conditions for growth.

### 2.5. Generation and Characterization of Rice OsBRP5 Silencing and Overexpressing Plants

**Figure 6:**
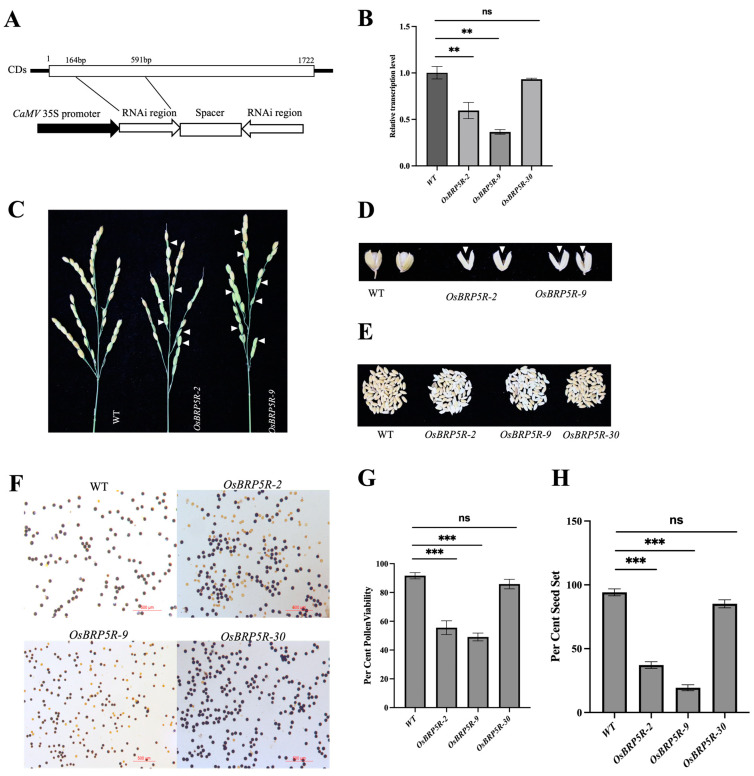
The silencing of OsBRP5 in rice affected pollen development and plant fruit setting. (**A**) Diagram of the OsBRP5-RNAi construct. Fragments containing the OsBRP5 segment in the sense and antisense orientation separated by an unrelated spacer were driven by the CaMV 35S promoter. (**B**) The transcription levels of OsBRP5 in WT and OsBRP5R plants. Error bars represent standard deviation (n = 3). (**C**) The panicles of WT and OsBRP5R plants. The green and empty green seeds are marked by arrows. (**D**) The mature seeds from wild-type and OsBRP5R plants. The empty seeds in OsBRP5R are highlighted by white arrows. (**E**) The total grains in the plant of OsBRP5R and WT rice. (**F**) I_2_-KI staining of mature pollen grains of WT and OsBRP5R plants. Bars = 500 μm. (**G**) The statistics for the pollen viability rate of WT and OsBRP5R plants. Data are shown as means ± SE (n = 3). (**H**) The statistics for the seed setting rate of WT and OsBRP5R plants. Data are shown as means ± SE (n = 6). Statistically significant differences are indicated by different symbols (** *p* < 0.01; *** *p* < 0.001. “ns” indicates no significant difference. one-way ANOVA with statistical significance using Student’s *t*-test).

Since OsBRP5 is homozygously lethal, we could not obtain a complete knockdown of OsBRP5. Therefore, we used RNAi and overexpression assays to investigate its biological function. Firstly, we synthesized a hairpin-like structure encompassing a modified RNAi segment derived from OsBRP5, under the control of the CaMV 35S promoter (Figure 6A). The vector for OsBRP5-RNAi was introduced into Nipponbare (*Oryza sativa* L.) callus through Agrobacterium-mediated transformation. Through the employment of PCR amplification, we have successfully identified 26 positive plants that bear the OsBRP5 RNAi cassettes among 32 transgenic plants. The relative expression levels of OsBRP5 experienced varying degrees of decline in most of the OsBRP5R (OsBRP5-RNAi) transformants via RT-qPCR analysis. For example, the relative expression levels of OsBRP5R-2 and OsBRP5-9 were 59.58% and 36.53%, significantly lower than that of WT plants, while the relative expression level of OsBRP5R-30 was 92.95%, similar to that of WT plants (Figure 6B).

After observing the growth and development phenotype, we found that OsBRP5R plants demonstrated unaltered vegetative growth patterns with respect to germination, tillering, and elongation. At the reproductive growth stage, we examined the panicles and noticed that OsBRP5R transformants had smaller panicles, and many seeds displayed a greenish coloration (Figure 6C). These green seeds were unable to achieve complete seed filling and remained hollow at the mature stage (Figure 6D). The number of unfilled grains in OsBRP5R was significantly higher compared to WT plants (Figure 6E). Subsequently, the fully developed pollen grains were immersed in a solution of I_2_-KI for staining purposes. Pollen grains lacking starch granules, which impedes their ability to absorb the I_2_-KI stain, are classified as nonviable, while those that exhibit staining are deemed viable (Figure 6F). The results showed that the pollen viability of OsBRP5R-2 and OsBRP5R-9 were 56.2% and 49.1%, respectively, significantly lower than that of WT plants (93.2%) (*p* < 0.05) (Figure 6G). At the mature stage, we analyzed yield-related traits and found that the seed-setting rate of OsBRP5R was significantly decreased in comparison to that of WT plants (*p* < 0.05) (Figure 6H). The seed-setting rate of OsBRP5R-2 and OsBRP5R-9 was 38.33% and 18.92%, while the seed-setting rate of WT plants was 94.00% (Figure 6H). The decreasing seed-setting rate resulted in a reduction in grain yield in the OsBRP5R plants. On the contrary, the relative expression level of OsBRP5R-30 was similar to that of WT plants. As a result, there were no significant differences in unfilled grains, pollen viability, or the seed setting rate between OsBRP5R-30 and WT plants (Figure 6H). The same methods were used to investigate the relative expression levels and growth and development phenotypes of overexpressing plants (Figure S1). It was found that overexpressing plants showed no abnormalities in growth and development compared to WT plants. The above results collectively indicate that OsBRP5 may affect seed setting by regulating pollen viability.

## 3. Discussion

As one of the world’s primary staple crops, rice is a critical source of sustenance for billions of people, playing a paramount role in global food security. However, it faces increasing challenges, including a growing population and climate change, which necessitate the development of high-yielding and stress-resistant rice varieties to meet food demands. Through the elucidation of gene functions, we can precisely target genes to enhance traits such as yield, quality, and stress tolerance in rice. Therefore, functional genomics research plays a pivotal role in improving rice and holds immense significance for global food security and sustainable agricultural development.

Transcription mediated by Pol II is a crucial process in the synthesis of mRNA for the majority of eukaryotic organisms, holding significant importance. General transcription factors (TFIIA, TFIIB, TFIID, TFIIE, TFIIF, and TFIIH) play a vital role in facilitating Pol II’s initiation of transcription at the correct genomic loci, operating through common promoter elements, such as the TATA box [13,14]. In the majority of eukaryotes, TFIIB commonly possesses two homologs that fulfill the role of general transcription factors for RNA polymerase I and III, respectively. In plants, however, the TFIIB-related protein family has undergone a substantial expansion, boasting a count of 14 members in *Arabidopsis* and 9 members in rice, respectively. 

BRP5/PTF2 proteins reside within a unique subfamily of TFIIB-related proteins, exclusive to the botanical and algal domains. Sequence analysis unveiled that the OsBRP5 protein comprises an N-terminal zinc ribbon domain and two cyclin fold core domains and belongs to the TFIIB superfamily (Figure 1A). These structural domains alone form the typical cyclin fold. Within the transcription complex, these structural domains span the C-terminal region of the TBP. This interaction is indispensable for the establishment of PIC [15,16]. Hence, TFIIB needs to interact with TBP to form the PIC. Therefore, by studying the interaction between TFIIB-related proteins and the TBP, we can garner a preliminary understanding of whether it continues to function as a general transcription factor or not. In this study, we employed Y2H and BiFC analyses to investigate the interaction between OsBRP5 and OsTBP. The results from both approaches consistently demonstrated that OsBRP5 does not interact with OsTBP1 or OsTBP2 but interacts with OsTBP3. In addition, the subcellular localization results showed that OsBRP5 was expressed in the nucleus. Taking these factors together, we postulate that it may still perform important functions as a general transcription factor.

A BLASTP search revealed that the rice OsBRP5 protein exhibits remarkable structural homology with the *Arabidopsis* AtBRP5 protein (Figure 1B). At the same time, it was found that AtBRP5 and OsBRP5 had a high sequence similarity in the C-terminal region, and this sequence was not identified. Moreover, these two genes were all related to reproductive growth. So, we speculated that the C-terminal region might play an important role in regulating reproductive growth. Prior studies have elucidated the interaction between AtBRP5 and AtTBP2, whereby AtBRP5 binds to the dsDNA [12]. A sequence comparison between AtBRP5 and OsBRP5 shows a high similarity in the two core domains and the C-terminal. Hence, we postulate that OsBRP5 exhibits functionally similar to B-type general transcription factors.

The temporal and spatial orchestration of gene expression governs pivotal developmental processes in plants, wherein gene regulation manifests at multiple hierarchical levels encompassing transcriptional, post-transcriptional, translational, and post-translational levels. Both website prediction and GUS stain experiments showed that the *OsBRP5* was mainly strongly expressed in reproductive organs such as florets, pistils, pollen grains, and anthers, indicating its potential role in reproductive growth.

The life cycle of angiosperms consists of two generations: the diploid sporophyte generation and the haploid gametophyte generation. Meiosis is a profoundly conserved process of division of diploid cells that generates haploid gametes. Within this intricate process, the sexually reproducing cells undergo a single round of DNA replication, followed by two consecutive rounds of nuclear division and, ultimately, form genetically recombined haploid generative cells [17,18]. The male and female gametophytes are also called pollen grains and embryo sacs, respectively. Pollen development in anther intricately involves gene expression and regulation. Mature fertile pollen can be produced with the regulation of hundreds of genes [19,20]. In rice, *OsMSP1* (*Multiple sporocytes1*), *OsTDL1A* (*Tapetum dertminant1-like1*), and *OsGAMYB* (*Gibberellic acid MYB transcription factor*) have been ascertained to be indispensable in early anther differentiation and development [21,22,23]. Post meiotic microspore development, the tapetum can provide nutrients for pollen development. *TDR* (*Tapetum degeneration retardation*) is preferentially expressed in the tapetum. In the anthers of a *tdr* mutant, the tapetum programmed cell death (PCD) was retarded, and the middle layer cells persisted. Consequently, the presence of such a mutation leads to abortive pollen development and complete male sterility [24]. In this research, we found that the decrease in *OsBRP5* gene expression leads to a decrease in pollen viability in rice, indicating its role in regulating pollen viability. However, the regulatory mechanism requires further research.

Previous studies have shown that *Arabidopsis AtBRP5* mutants that have a T-DNA insertion in the 5′-UTR could lead to pollen germination failure, embryo development disruption, and seed abortion. Therefore, homozygous frameshift *AtBPR5* mutations cannot be inherited by offspring. Only heterozygous frameshift mutations can be passed on to the next generation. In our research, we obtained *OsBRP5* transgenic rice plants containing a frameshift mutation using CRISPR/Cas9 technology. However, unlike *Arabidopsis atbrp5* mutants, *brp5/ptf2* frameshift mutations are not passed on to offspring in rice. The silencing of rice *BRP5/PTF2* expression had little effect on vegetative growth and panicle formation but strongly affected pollen development and grain formation, as found in the *Arabidopsis atbrp5* knockdown mutant. These results suggest that *Osbrp5/ptf2* assumes a crucial function in the development or viability of both male and female gametes. The above research emphasizes both the evolutionary conservation of this gene’s function and the distinctions between monocotyledonous and dicotyledonous plants.

This study establishes a foundation for deeper investigations into the gene function and regulatory mechanisms of *OsBRP5* in rice. It also offers new genetic resources and innovative approaches for rice enhancement. As the next step, we will mine the candidate target genes that directly interact with *OsBRP5* with the help of Omics methods, such as RNA-seq and ChIP-seq, to unravel the new mechanisms governing pollen viability. Additionally, we will employ immunoprecipitation to isolate the nuclear protein complex and employ proteome sequencing to identify its components, thus gaining a clear understanding on whether OsBRP5 continues to function as a general transcription factor.

## 4. Materials and Methods

### 4.1. Plant Materials

Rice mutants and wild-type (WT) plants were all based on the background of *the japonica rice* (*Oryza sativa* Nipponbare var.). Rice plants were cultivated hydroponically in an adapted rice culture solution comprising 0.998 mM CaCl_2_, 1.643 mM MgSO_4_, 0.009 mM MnCl_2_, 0.075 mM (NH_4_)6Mo_7_O_24_, 0.019 mM H_3_BO_3_, 0.155 mM CuSO_4_, 1.425 mM NH_4_NO_3_, 0.2 mM NaH_2_PO_4_, and 0.513 mM K_2_SO_4_, with a pH range of 5.2–5.5 [25]. Rice plants were cultivated in a controlled growth chamber, with a photoperiod of 13 h of daylight (30 °C) followed by 11 h of night-time (23 °C). The photon density was kept at approximately 200 μmol·m^−2^s^−1^, while the humidity level was maintained at around 60%, as described in previous studies [26]. Tobacco (*Nicotiana Benthamiana* var.) plants were grown under a standard light regime of 12 h of light and 12 h of darkness, at a temperature of 25 °C and with a relative humidity level of 70%. One-month-old seedlings were used for injection.

### 4.2. Protein Sequence Analysis

The protein sequences and corresponding gene sequences of 9 rice and 14 *Arabidopsis* TFIIB-related proteins were retrieved from the Rice Genome Annotation Project database (RGAP) (https://rice.plantbiology.msu.edu/ (accessed on 21 September 2022)) and the *Arabidopsis* Information Resource (https://www.arabidopsis.org/ (accessed on 21 September 2022)), respectively. The chromosomal localization, amino-acid length, full-length cDNA accessions, molecular weight (MW), and isoelectric point (pI) of *OsBRP5* (*LOC_Os08g42020*) were obtained from RGAP, too. Sequence analyses were conducted using the InterPro Program (http://www.ebi.ac.uk/interpro/ (accessed on 1 September 2023)). The evolutionary relationships between the TFIIB gene families were identified via comparing the protein sequences of TFIIB-associated proteins in Arabidopsis and rice using Clustal W with default parameters [27]. The neighbour-joining (NJ) method was used to construct the phylogenetic trees in MEGA 6.0. The Poisson correction model, pairwise deletion, and bootstrap test with 1000 replications were used to test the statistical reliability [28].

### 4.3. GUS Staining

In order to produce the *OsBRP5*-GUS plasmid, the complete *OsBRP5* coding sequence (CDS) was amplified through PCR and then directionally inserted into the pCAMBLA1300-GUSPlus1827 vector, employing the *Kpn I* and *Hind III* restriction sites. Introduction of recombinant plasmids into rice callus via *A. tumefaciens*-mediated transformation methods and GUS activity assays were conducted according to the method described by Lee [29], with a minor modification. Tissue samples derived from *OsBRP5*-GUS transgenic plants were submerged in cold acetone (90%) at −20 °C for 20 min. We proceeded by rinsing to remove the remaining traces of acetone with distilled water. The tissue specimens were then immersed in GUS staining solution comprising 2 mM 5-bromo-4-chloro-3-indolyl β-D-glucuronide (X-Gluc), 10 mM EDTA, 2 mM potassium ferricyanide, 2 mM potassium ferricyanide, and 0.1% Triton X-100 in a 50 mM sodium phosphate buffer with a pH of 7.0. Following a 20 min vacuum treatment, the tissue was subjected to overnight incubation at 37 °C. Upon completion of the staining process, the samples underwent successive bleaching with 75% ethanol at hourly intervals until the chlorophyll pigment was completely eradicated. GUS staining was observed under a microscope (Leica, Wetzlar, Germany).

### 4.4. Subcellular Localization

Total RNAs were isolated from Nipponbare (*Oryza sativa* L.) with Trizol reagent (Tiangen, Beijing, China). RNAs were reverse-transcribed to cDNAs using the Reverse Transcription Kit (Vazyme, Nanjing, China) and served as the templates. Specific primers were designed to clone the CDS of *OsBRP5* without a termination codon (Table S1). Subsequently, this sequence was ligated into a 1300UR-sGFP vector employing the *Spe I* and *Bam HI* restriction sites. The recombinant plasmid was transformed into *A. tumefaciens* strain GV3101 via gene importer (Bio-Rad, Hercules, CA, USA). The recombinant vectors *OsBRP5*-GFP and Ubi-GFP (used as a control) were transfected into one-month-old *N. benthamiana* plants that already expressed a nuclear marker H2B-RFP protein via employing 1 mL syringes without needles. After incubation at 25 °C for 36–48 h, a confocal laser scanning microscope (Nikon, Tokyo, Japan) was used to observe the green fluorescence signal and collect images [30].

### 4.5. Yeast Two-Hybrid (Y2H) Screen and Bimolecular Fluorescence Complementation (BiFC) Assay

The Y2H assays were conducted utilizing the Gal4 vector system (Clontech, Mountain View, CA, USA, www.clontech.com/). Using the japonica rice (*Oryza sativa* Nipponbare var.) as a template, the complete *OsBRP5* CDS was amplified through PCR and then directionally inserted into the pGADT7 vector (Clontech, Mountain View, CA, USA) employing the *EcoR I* and *Bam HI* restriction sites. The CDS of *OsTBP1-OsTBP3* was cloned and ligated into the pGBKT7 vector using specific primers for fusion to the GAL4 DNA binding domain, respectively (Table S1). In order to ascertain their interaction, we introduced the fusion constructs alongside the control plasmids AD (activation domain) and BD (binding domain) into AH109 yeast cells utilizing the lithium acetate-mediated technique [31]. The positive control for the interaction was established utilizing pGBKT7-53 and pGADT7-T, while the negative control was established using the empty pGBKT7-Lam and pGADT7-T vector. The transformed cells were adjusted to an OD_600_ of about 0.3 and cultivated on SD/-Leu-Trp (SD/2) plates for 3–4 d at 30 °C. Then, 3–5 monoclones were selected from the SD/2 picking medium in 500 μL of sterilized double-distilled water (ddH_2_O) and diluted to 10-3 on a gradient scale and sequentially spot-coated on SD/-Trp/-Leu/-Ade/-His (SD/4) medium, and incubated at 30 °C for 3–7 d. 

For the BiFC assay, the carrier was constructed using the ligase-independent cloning (LIC) method followed by Aslanidis [32]. The CDS of *OsBRP5* and *OsTBP1-OsTBP3* were cloned into pCV-nYFP and pCV-cYFP vectors with specific primers, respectively (Table S1). The negative controls, namely, pCV-cYFP/OsBRP5-NY and OsTBP-CY/pCV-nYFP, were employed in this study. The recombinant green fluorescence signals emitted by *N. benthamiana* were scrutinized in accordance with the previously established method [33]. A confocal laser scanning microscope (Nikon/C2) was used to detect fluorescence signals and capture images 36–48 h after conversion.

### 4.6. CRISPR/Cas9 Gene Editing and Phenotype Observation

Construction of CRISPR/Cas9 gene editing vectors was performed as previously described [32]. Two different sites on the exon of *OsBRP5*, both located near the transcriptional start site of the *OsBRP5* CDs, were carefully identified as targets for genome editing (Table S1). Two target sequences were incorporated into sgRNA expression cassettes through overlapping PCR, generating pU3-*OsBRP5*-sgRNA and pU6a-*OsBRP5*-sgRNA fragments. Subsequently, these fragments were cloned into pYLCRISPR/Cas9Pubi using restriction–ligation reactions involving *Bsa I* and T4 DNA ligase, resulting in the creation of the pCRISPR-*OsBRP5* constructs. Then, the plasmids were introduced into *A. tumefaciens* strain EHA105 via electroporation. Subsequently, the rice callus underwent a transformation process according to the previously outlined procedure [33]. To identify mutations on the target locus, PCR amplification was performed utilizing primers flanking the region of the two targets of *OsBRP5* in T_0_ transgenic lines (Table S1). Subsequently, direct sequencing was performed to determine the sequence composition. The growth and development phenotypes of mutants were compared with WT plants. Seeds were harvested in T_0_ generation, followed by germination, and the segregation of mutant lines in T_1_ generation was subsequently analyzed.

### 4.7. RNAi and Overexpression of OsBRP5 and Phenotype Observation

In order to produce the RNAi vectors targeting *OsBRP5*, we amplified a 427 bp fragment near the transcription start site from the exonic region of *OsBRP5* and carefully inserted it in both the sense and antisense orientations into the RNAi vector pTCK303, thereby fashioning a hairpin structure. After sequencing, the recombinant *OsBRP5*-RNAi vector was obtained. Since the recombinant 1300UR-sGFP vector is driven by the maize ubiquitin promoter, it is also used as an overexpression vector (*OsBRP5*-OE). Subsequently, these vectors were introduced into the *A. tumefaciens* strain EHA105 and transformed into the rice callus [33]. The primers involved in plasmid construction are provided in Table S1. After the obtaining of transgenic plants, DNA was extracted and PCR amplification was used to detect the presence of the recombinant plasmid with specific primers (Table S1). Then, qRT-PCR was used to determine the relative expression levels of *OsBRP5* in RNAi and overexpression plants. After that, the phenotypes of RNAi and OE plants were observed using WT rice as a control. The measured traits included plant height, spike length, pollen viability, and seed setting rate. Pollen viability was detected using iodine–potassium iodide (I_2_-KI) staining, according to Prasad [34]. Pollen iodine staining was carried out on spikelets nearing the blooming stage, and 3 spikelets were selected from each spike for microscopic examination. The number of iodine-stained pollens was counted, and the proportion against total pollen grains was computed as the pollen viability ratio. A minimum of 300 pollen grains per sample was meticulously examined under the microscope. Images were observed using a Leica microscope and captured with Leica Application Suite software 4.4.0 (Leica, Wetzlar, Germany). Pollen grains that exhibited a substantial size, spherical shape, and distinct staining were classified as fertile, whereas those that appeared diminutive, flattened, and lacked staining were deemed infertile. After harvest, the seeds were dried at 28 °C for 5 d, and the seed setting rate was calculated. The seed setting rate was measured using rice grains collected from three spikelets per plant. The seed setting rate was estimated as followed: seed setting rate% = plump grain number/total grain number × 100%.

### 4.8. RNA Isolation, cDNA Synthesis, and qRT-PCR Analysis

In order to explore the relative expression levels of OsBRP5-RNAi and OsBRP5-OE lines, total RNAs were extracted from rice leaves utilizing the TRIzol reagent (Tiangen, Beijing, China). RNAs were subjected to reverse transcription, converting them into cDNAs, using the RNA reverse-transcription kit equipped with gDNA Remover (Vazyme, Nanjing, China). qRT-PCR was conducted utilizing TB Green Premix Ex TaqTM II (Takara, Kusatsu, Japan) via a qTOWER 3G Real-time PCR system (Analytic Jena, Jena, Germany). The reaction mixture comprised 10 μL TB Green Mix, 8.4 μL ddH_2_O, 0.6 μL primers, and 1 μL cDNA. The thermal cycling parameters were as follows: an initial denaturation step at 95 °C for 1 min, followed by 45 cycles of denaturation at 94 °C for 10 s, and annealing at 55 °C for 15 s. β-actin served as the internal control, and the relative expression levels were determined utilizing the 2^−ΔΔCt^ method [35]. All the experiments were replicated biologically thrice. The complete list of primers can be found in Table S1.

### 4.9. Statistical Analysis

In this research, we have performed one-way ANOVA (analysis of variance) for multiple pairwise comparisons using GraphPad Prism 8.0 (GraphPad Prism Software Inc., San Diego, CA, USA). Statistical significance was analyzed via Student’s *t*-test using SPSS 22.0 (SPSS, Chicago, IL, USA). All trials were conducted with three biological replicates. The results are depicted as mean ± SE in the figures, while ** and *** represent significant differences at *p* < 0.01 and *p* < 0.001, respectively.

## 5. Conclusions

In this study, we have discovered OsBRP5, a transcription factor associated with pollen development in rice. OsBRP5 promoter-driven β-glucuronidase expression was present exclusively in stamen and reproductive organs. Subcellular localization showed that OsBRP5 was targeted to the nucleus. The interaction analysis results showed that OsBRP5 interacted with TBPs both in vitro and in vivo, indicating that it might still perform important functions as a general transcription factor. Using CRISPR/Cas9, we have generated transgenic rice plants containing loss-of-function frameshift mutations in the OsBRP5/PTF2 gene and discovered that the mutations were not transmitted to progeny in rice. The silencing of rice BRP5/PTF2 expression had little effect on vegetative growth and panicle formation but strongly affected pollen development and grain formation, as found in the Arabidopsis atbrp5 knockdown mutant. These results support the theory that plant-specific BRP5/PTF2 has evolved to play a critical and specific role in plant sexual reproduction.

## Data Availability

No new data were created or analyzed in this study. Data sharing is not applicable to this article.

## Supplementary Materials

The following supporting information can be downloaded at: https://www.mdpi.com/article/10.3390/ijms242216473/s1.
