# Supplementary material for: TFIIB-Related Protein BRP5/PTF2 Is Required for Both Male and Female Gametogenesis and for Grain Formation in Rice
Source: Int J Mol Sci. 2023 Nov 18;24(22):16473. doi: 10.3390/ijms242216473 (PMC10671200; doi:10.3390/ijms242216473)
Supplement: Supplementary file 1 [file ijms-24-16473-s001.zip › Figure S1 Construction and expression detection of OsBRP5 overexpression vector in rice.pdf]

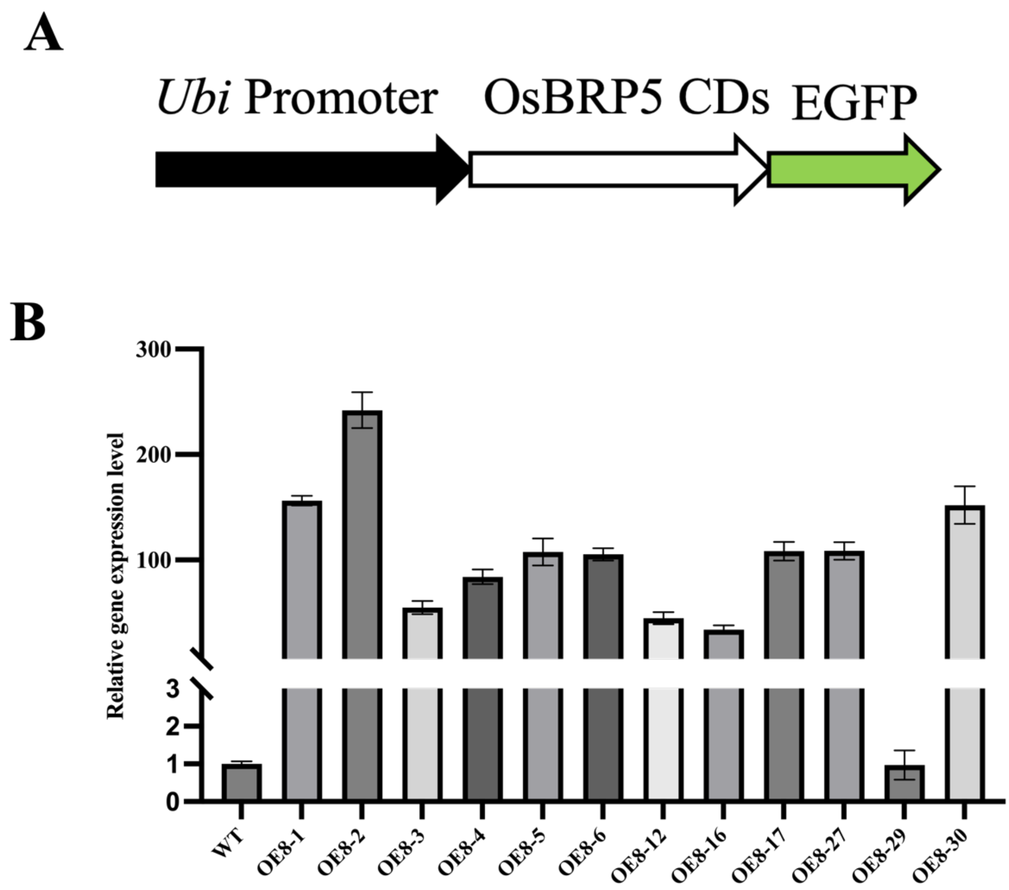

Figure S1 Construction of *OsBRP5* overexpression vector and relative expression levels of *OsBRP5* in rice. (A) Diagram of the *OsBRP5-OE* construct. Fragments containing the *OsBRP5* CDs driven by the *Maize* Ubiquitin promoter; (B) Relative expression levels of *OsBRP5* in WT and OsBRP5 overexpressing plants.
